# Changes in trachoma indicators in Kiribati with two rounds of azithromycin mass drug administration, measured in serial population-based surveys

**DOI:** 10.1371/journal.pntd.0011441

**Published:** 2023-07-07

**Authors:** E. Brook Goodhew, Raebwebwe Taoaba, Emma M. Harding-Esch, Sarah E. Gwyn, Ana Bakhtiari, Robert Butcher, Anasaini Cama, Sarah Anne J. Guagliardo, Cristina Jimenez, Caleb D. Mpyet, Kab Tun, Karana Wickens, Anthony W. Solomon, Diana L. Martin, Rabebe Tekeraoi

**Affiliations:** 1 VAAS Contractors, Atlanta Georgia; 2 Ministry of Health and Medical Services, South Tarawa, Kiribati; 3 Clinical Research Department, London School of Hygiene & Tropical Medicine, London, United Kingdom; 4 Centers for Disease Control and Prevention, Atlanta Georgia; 5 International Trachoma Initiative, Task Force for Global Health, Atlanta, Georgia; 6 The Fred Hollows Foundation, Melbourne, Australia; 7 Sightsavers International, London United Kingdom; 8 Department of Ophthalmology, College of Health Sciences, University of Jos; Jos, Nigeria, and Sightsavers, Nigeria Country Office, Kaduna, Nigeria; 9 Oak Ridge Institute for Science and Education, Oak Ridge, Tennessee; 10 Department of Control of Neglected Tropical Diseases, World Health Organization, Geneva, Switzerland; RTI International, UNITED REPUBLIC OF TANZANIA

## Abstract

Baseline mapping in the two major population centers of Kiribati showed that trachoma was a public health problem in need of programmatic interventions. After conducting two annual rounds of antibiotic mass drug administration (MDA), Kiribati undertook trachoma impact surveys in 2019, using standardized two-stage cluster surveys in the evaluation units of Kiritimati Island and Tarawa. In Kiritimati, 516 households were visited and in Tarawa, 772 households were visited. Nearly all households had a drinking water source and access to an improved latrine. The prevalence of trachomatous trichiasis remained above the elimination threshold (0.2% in ≥15-year-olds) and was virtually unchanged from baseline. The prevalence of trachomatous inflammation—follicular (TF) in 1–9-year-olds decreased by approximately 40% from baseline in both evaluation units but remained above the 5% TF prevalence threshold for stopping MDA. TF prevalence at impact survey was 11.5% in Kiritimati and 17.9% in Tarawa. Infection prevalence in 1–9-year-olds by PCR was 0.96% in Kiritimati and 3.3% in Tarawa. Using a multiplex bead assay to measure antibodies to the *C*. *trachomatis* antigen Pgp3, seroprevalence in 1–9-year-olds was 30.2% in Kiritimati and 31.4% in Tarawa. The seroconversion rate, in seroconversion events/100 children/year, was 9.0 in Kiritimati and 9.2 in Tarawa. Seroprevalence and seroconversion rates were both assessed by four different assays, with strong agreement between tests. These results show that, despite decreases in indicators associated with infection at impact survey, trachoma remains a public health problem in Kiribati, and provide additional information about changes in serological indicators after MDA.

## Author summary

This study compares different indicators for the eye disease trachoma before and after two annual rounds of mass azithromycin treatment of affected communities of Kiribati. We saw decreases in the proportion of children with the sign TF (trachomatous inflammation—follicular) that is used by trachoma programs to make decisions about starting and stopping mass treatment, but these decreases were not sufficient to make the decision to stop. We also saw decreases in infection and antibody levels after treatment. The study provides important public health information to Kiribati trachoma programs, but also adds to the growing body of evidence to guide the use of alternative indicators–infection and antibody–to support these programs. Measuring antibody levels in communities over time helps us understand how antibody levels change as community infection prevalence decreases. Understanding antibody dynamics over time can help us better estimate antibody-based thresholds for surveillance after elimination of trachoma as a public health problem.

## Introduction

Trachoma is the leading infectious cause of blindness. In June 2022, it was considered a public health problem in 44 countries [1]. Elimination of trachoma as a public health problem relies on the SAFE strategy: Surgery to correct eyelid deformation causing eyelashes to touch the eyeball (trachomatous trichiasis or TT) [2], mass drug administration of Antibiotics to clear conjunctival infection with the causative agent *Chlamydia trachomatis*, and efforts to address Facial cleanliness and Environmental improvement to control spread of conjunctival *C*. *trachomatis* [3]. Implementation of the AFE components of SAFE is based on prevalence of the sign trachomatous inflammation—follicular (TF) ≥5% in children aged 1–9 years in a population (typically called an evaluation unit [EU]), defined as an administrative health district of 100,000–250,000 persons) [4]. TF prevalence in this age group is intended to reflect recent ocular *C*. *trachomatis* transmission intensity [5]. Controlling infection in children should lead to fewer cases of TT and blindness later in life, but TT may take decades to develop. TT prevalence in adults is therefore an indicator that lags many years behind the prevalence of TF and conjunctival *C*. *trachomatis* infection in children [6].

Baseline trachoma mapping conducted in Kiribati in 2015–2016 showed >30% TF prevalence in 1–9-year-olds, as well as TT prevalence >0.2% (the elimination threshold for TT) in ≥15-year-olds [7,8]. Testing for infection and anti-*C*. *trachomatis* antibody was also done. More than 25% of children with TF on the island of Kiritimati were infected, and the infection prevalence in Tarawa was 27.4% among 1–9-year-olds. In both sites, >50% of children were antibody positive at those baseline surveys.

There are several studies highlighting the potential programmatic value of including additional indicators in trachoma surveys, particularly infection and antibody testing [7–9]. Antibody testing can potentially provide information on transmission intensity of ocular *C*. *trachomatis* by integrating seroprevalence with age. Repeated infection is needed to develop the conjunctival scarring that can lead to TT, corneal opacity, and blindness [10], providing a more complete picture of transmission than infection prevalence. The interpretation of serology will be facilitated by understanding the dynamics of antibody responses over time, using repeated sampling [11].

In Kiribati, following the baseline surveys outlined above, the Ministry of Health and Medical Services undertook two rounds of azithromycin mass drug administration (MDA) of all residents, and in 2019 conducted trachoma impact surveys (TIS) in Tarawa and Kiritimati Island. We took advantage of the fact that Kiribati’s baseline surveys had included infection and antibody testing to compare these indicators before and after the two MDA rounds. To minimize potential variations in antibody data due to testing at different sites, we repeated antibody testing of baseline dried blood spot (DBS) specimens at the U.S. Centers for Disease Control and Prevention (CDC), side-by-side with the TIS specimens. This paper reports on the results of the 2019 TIS in two EUs (Tarawa and Kiritimati Island) in Kiribati. We also compare serological data generated by three different antibody tests (multiplex bead assay [MBA], ELISA, and lateral flow assay [LFA]) at TIS and evaluate changes in serologic parameters between the 2015–2016 baseline surveys [7,8] and 2019 TIS using MBA and ELISA.

## Methods

### Ethics

Ethical approval for TIS was granted by the Kiribati Ministry of Health and Medical Services (25/05/16) and the London School of Hygiene & Tropical Medicine (LSHTM, reference 11158). Tropical Data has ethics approval from LSHTM (16105) to support health ministries to conduct trachoma prevalence surveys. Written informed consent for specimen collection from participants aged 1–9 years was obtained from parents or guardians. CDC staff did not interact with study participants or have access to identifying information and were considered to be non-engaged in research. Details on ethical considerations for the baseline surveys are found elsewhere [7,8].

### Study sites

**Fig 1:**
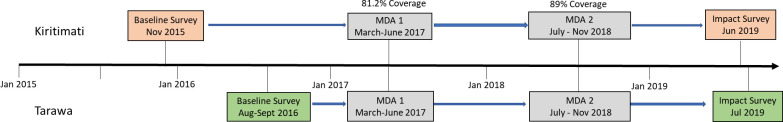
Survey and mass drug administration (MDA) timelines.

Kiribati has a population of approximately 118,000, found on islands spread across 1.4 million square miles of ocean. Baseline surveys [7,8] and TIS were conducted in two EUs comprising the most populous areas of the country: the Tarawa atoll (Betio, South Tarawa, and North Tarawa) and Kiritimati Island. Baseline mapping was conducted in November 2015 on Kiritimati and in August–September 2016 on Tarawa. TIS were conducted in both sites in June–July 2019. In Tarawa, we used a two-stage cluster sampling design of 30 first-stage clusters. On Kiritimati, we selected 21 clusters; the population and the total number of villages here is much smaller than in most trachoma-endemic EUs, and an adapted sampling strategy was used [8]. In this strategy, all four inhabited villages (the smallest administrative unit) on Kiritimati were visited, with the number of households to be invited to participate in each village proportional to that village’s relative size. All residents in selected households aged ≥1 year were invited to participate. Everyone aged ≥1 year who consented to participate had eye exams conducted; 1–9-year-olds also had DBS (from finger-prick) and ocular conjunctival swabs collected. Household heads responded to questions about water, sanitation, and hygiene access. A timeline of the surveys is shown in Fig 1.

### Clinical grading

Eye exams were performed by Tropical Data-certified graders using the World Health Organization (WHO) simplified grading scheme to assess for signs of trachoma (TT, TF and trachomatous inflammation—intense [TI]) [2]. TI data were collected as per current survey recommendations [12]but are not reported here. Participants with TT were additionally graded for the presence of trachomatous scarring (TS). TF was defined as the presence of 5 or more follicles, each at least 0.5 mm in diameter, in the central part of the upper tarsal conjunctiva of one or both eyes. Follicle size guides [13] were used during grading. People with TF or other evidence of conjunctivitis were given 1% topical tetracycline eye ointment. TT was defined as upper and/or lower eyelashes touching the eye (studies were planned prior to the 4^th^ Global Scientific Meeting for Trachoma, at which the definition of TT was amended). People with TT were referred to a health clinic for surgery. Data were recorded by Tropical Data-certified recorders on Android smartphones and subsequently transferred to a cloud-based server.

### DBS collection

Finger-prick blood was collected onto filter paper wheels with circular extensions calibrated to hold 10 μL of blood (TropBio Pty Ltd., Townsville, Queensland, Australia). Filter papers were dried overnight, placed in sealable plastic bags with desiccant, and stored at -20°C until being shipped to CDC, Atlanta, at ambient temperature. DBS were then stored in a -20°C freezer until processing.

### Conjunctival swab collection

The grader passed a sterile cotton swab three times over the everted right conjunctiva with a 120°rotation between each pass. Ocular swabs were immediately placed in a dry, sterile tube and placed into a container with ice while in-field. Each night upon return from the field, swabs were placed in the laboratory freezer. Swabs were shipped on ice packs to CDC, Atlanta, and immediately stored at -20°C upon arrival in the laboratory.

### Serologic testing

**Fig 2:**
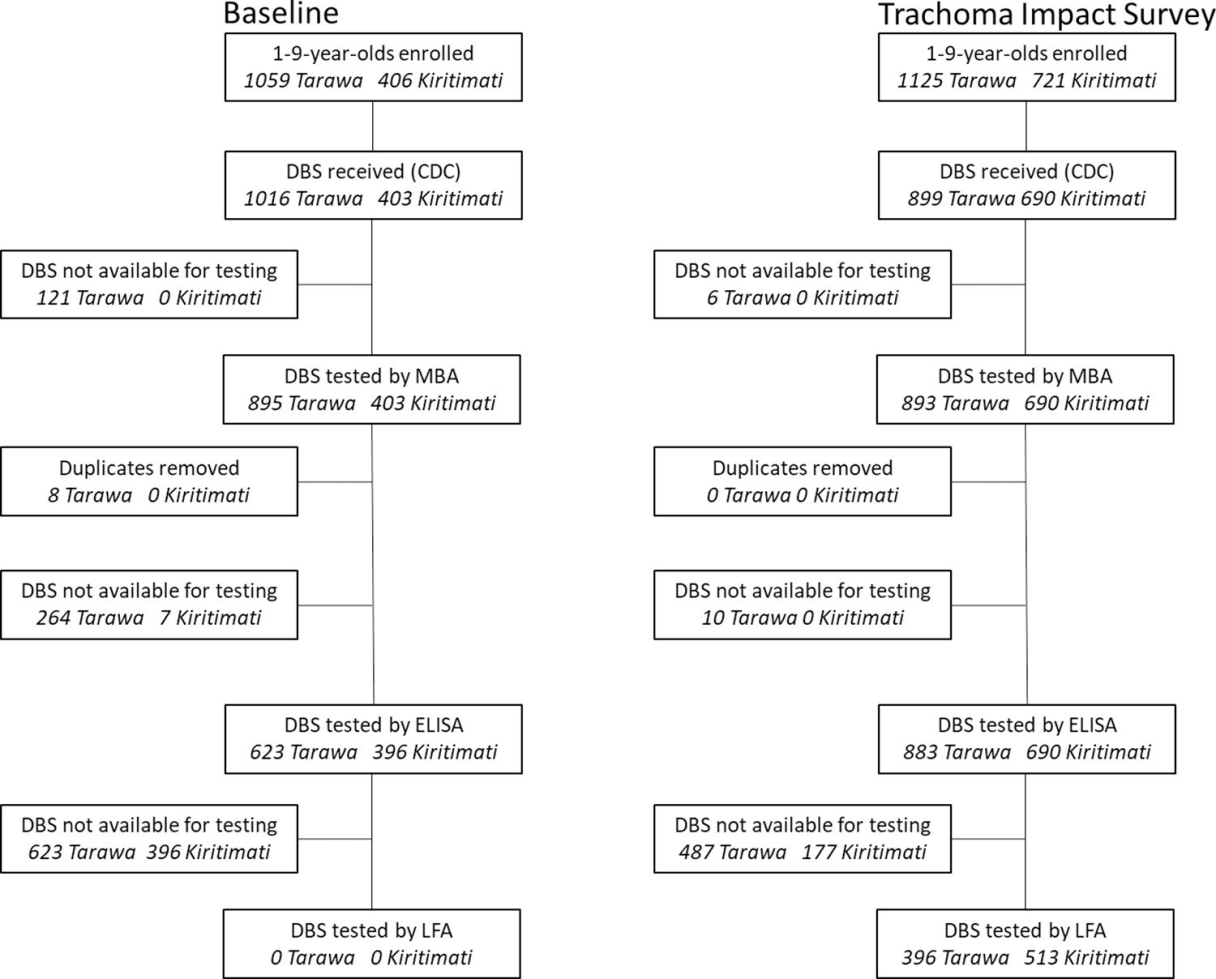
Flow chart for dried blood spot (DBS) testing.

Antibodies against the *C*. *trachomatis* antigen Pgp3 were detected using MBA and ELISA for specimens collected at baseline, and by MBA, ELISA, and LFA for specimens collected at TIS, because there were insufficient DBS specimens remaining to run the baseline specimens on LFA (Fig 2). MBA data were also collected on antibodies to CT694 at baseline and TIS. All antibody testing was done in CDC laboratories in Atlanta, GA, USA. Both baseline and impact specimens were tested concurrently using the same technician, assay reagents, and equipment to minimize variability.

### Multiplex bead assay (MBA)

DBS extensions were prepared and tested as previously described [14]. Briefly, serum was eluted from extensions overnight at 4°C in sample buffer (1X PBS, 0.5% casein, 0.5% polyvinyl alcohol, 0.8% polyvinylpyrrolidone, 0.3% Tween 20, 0.02% NaN_3_) containing 3 μg/mL *Escherichia coli* extract (Buffer B) and diluted to a final concentration of 1:400. Antigen-coupled beads (Pgp3 and Ct694) were incubated with diluted sample for 1.5 hours then washed with 0.05% Tween-20 in PBS (PBST). Beads were washed with PBST and incubated with biotinylated mouse anti-human IgG and biotinylated mouse anti-human IgG4 for 45 minutes to detect IgG bound to Pgp3 and Ct694 on the beads. After additional washes with PBST, beads were incubated for 30 minutes with phycoerythrin (PE)-labeled streptavidin to detect bound biotinylated anti-human IgG. After detection, loosely bound antibodies were removed with a final 30-minute incubation in PBST containing 0.5% BSA and 0.02% NaN_3_, washed, resuspended in PBS, and stored overnight at 4°C.

The next day, plates were read on a Bio-Plex 200 instrument (Bio-Rad, Hercules, CA) equipped with Bio-Plex manager 6.0 software (Bio-Rad). The median fluorescence intensity (MFI) with background signal (Buffer B alone) subtracted out (MFI-bg) was recorded for each antigen for each sample.

### Enzyme-linked immunosorbent assay (ELISA)

Immulon 2HB plates (Thermo Fisher Scientific, Waltham, MA) were coated with 300 ng/mL of Pgp3 antigen in NaCO_3_ pH 9.6 overnight at 4°C. The next day, wells were washed 4 times with 0.3% Tween-20 in 1X PBS (PBST2) and incubated for 30 minutes with 100 μL of StabilCoat (SurModics, Eden Prairie, MN, USA). Buffer was decanted from wells, and plates were dried in a vacuum oven at 30–40°C for 4 hours. Dried plates were stored in foil packages with a 1 g of desiccant at 4°C until use. Each DBS extension was eluted overnight at 4°C in 250 μL PBST2 containing 5% milk powder (PBST-milk) for a final serum dilution of 1:50. Diluted sample (50 μL) was added to each well of a Pgp3 coated ELISA plate and incubated for 2 hours. Wells were washed 4 times with 200 μL of PBST2. Pgp3-conjugated to horseradish peroxidase (Expedeon, San Diego, CA) was diluted 1:1,000 in PBST and 50 μL was added to each well to detect any bound anti-Pgp3 immunoglobulin. After a 1-hour incubation, plates were washed 4 times in 200 μL of PBST2. 3,3’, 5,5’ tetramethylbenzidine (TMB) was added (50 μL/well) and incubated for 10 minutes. To stop the reaction, 50 μL 1M H_2_SO_4_ was added per well. The plates were read immediately at 450 nm on a microplate reader. A set of standards (1000U, 500U, 200U, 50U) along with negative human serum (NHS) and a blank (PBST-milk) were run on each plate. The optical density at 450 nm (OD450) of the blank well was subtracted from the OD450 value of each sample (OD450-bg). The OD450-bg value of each sample was normalized to the 500U standard (Norm OD450-bg) [15].

### Lateral flow assay using black latex (LFA-latex)

Pgp3-LFA dipsticks were manufactured and used to test DBS as previously described [16] and the same lot used for the entire study following quality assurance with external positive and negative controls. Samples were tested by the LFA-dipstick assay using a black latex detector reagent. Pgp3-latex (Expedeon) and SA-gold (Arista Biologicals) were diluted 1:240 and 1:120, respectively, in PBST2 (0.3% Tween-20 in PBS) to create a conjugate mastermix. Each DBS was eluted in 60 μL of conjugate mastermix in a well of a flat-bottom 96-well plate and incubated overnight at 4°C. Pgp3 LFA-dipsticks were added to each well and incubated for 15 minutes, until all liquid was absorbed. PBST (80 μL) was then added to each well to clear background caused by hemolyzed red blood cells on the nitrocellulose membrane. After background clearance (approximately 5 minutes) each LFA was scored as positive, negative, or invalid.

### Cutoff values

Receiver operator characteristic (ROC) curve analysis was used to calculate the MBA positivity cutoff with a panel of 77 Pgp3 IgG antibody positive individuals [16] and 74 chlamydial microimmunofluorescence assay-negative USA pediatric samples. A cutoff value of 850 MFI-bg was established for Pgp3 and 146 MFI-bg for Ct694, with an indeterminate range of 736 to 963 and 140 to 152, respectively [17]. An ELISA positive cutoff value of 0.1525 OD 450 nm (Norm A-blank) was calculated using a finite mixture model to classify the samples as seropositive or seronegative based on maximum likelihood methods. The threshold was set at the mean of the Gaussian distribution of the seronegative population plus four standard deviations to ensure high specificity [18].

### Infection testing

Ocular swabs were tested using a Cepheid GeneXpert CT/NG kit [19]. Swabs were eluted in 1 mL of diethyl pyrocarbonate (DEPC) water for >1 hour at 4°C. 200 μL of each specimen was added to a Cepheid transport media tube, then 260 μL of this solution was added to a 2 mL tube to create pools of 5 specimens each. 1200 μL of each pool was added immediately to the sample chamber of the Cepheid CT/NG cartridge and run according to manufacturer’s instructions. Samples that were part of each positive pool were subsequently tested individually to identify positive specimens. Each run included a sample processing control, sample adequacy control, and probe check control included with each kit. Pools and specimens from invalid runs were re-tested.

### Statistical analysis

Statistical analyses were conducted in R (version 3.6.3) or Graphpad Prism Quickcalcs. TF prevalence estimates were calculated using the mean of the age-standardized cluster-level TF proportions, a standard method used by Tropical Data (https://github.com/itidat/tropical-data-analysis-public) and by its predecessor, the Global Trachoma Mapping Project [12]. Trichiasis prevalence estimates were similarly adjusted, for age and gender. We calculated 95% confidence intervals (CIs) by bootstrapping the cluster means over 10,000 iterations. Age-standardized EU-level seroprevalence proportions were used to generate overall seroprevalence estimates for 1–9-year-olds. Seroconversion rates (SCRs) and associated credible intervals were calculated using Bayesian serocatalytic models, which characterize the change in proportion of seropositive individuals by age [20–22], as previously described [22]. Following methods from Pinsent et al (22), we evaluated two transmission scenarios for each dataset 1) a constant rate of transmission (Model 1) and 2) a drop in transmission at a fixed point in time (Model 2). An informative prior was used to estimate the seroreversion rate, rho (Pgp3 ~ N(0.26, 0.003); ct694 ~ N(0.017,0.002)), based on previously published work. A Gelman-Rubin statistic (GR) <1.1 and an effective sample size (ESS) >300 were used to ensure chain convergence. If both models produced acceptable GR and ESS, the model with the lowest Deviance Information Criterion (DIC) was selected as the most likely scenario of the observed data. Seroconversion rates (SCR) were scaled to number of events per 100 people per year for ease of interpretation. Age-standardized EU-level infection prevalence proportion were used to generate overall infection prevalence estimates for 1–9-year-olds Prevalence ratios and 95% confidence intervals of the change between impact and baseline in 1–3-year-olds were calculated for each assay and island by log-binomial regression. Values were then transformed via the equation relative change = 100*(*PR– 1)*.

## Results

### Trachoma impact survey demographic information

There were 772 households surveyed in Kiritimati and 516 households surveyed in Tarawa (Table 1). In both EUs, the majority of households had improved drinking water sources, access to a water source within 30 minutes, and improved latrines (Table 1). In Kiritimati, 872 1–9-year-olds were enumerated, and 764 1–9-year-olds were examined for TF; age-adjusted TF prevalence was 11.5% (95% CI: 8.6%-14.1%). In Tarawa, 1243 1–9-year-olds were enumerated, and 1002 1–9-year-olds were examined for TF; age-adjusted TF prevalence was 17.9% (95% CI 14.7% –21.8%). The previously-published baseline survey data are provided in Table 2 for comparison.

**Table 1:** Trachoma Impact Survey Demographic Information.

	Kiritimati	Tarawa
**# clusters**	21	30
**# households (HH)**	516	772
**1–9 year-olds enumerated**	872	1243
**1–9 year-olds surveyed (% of enumerated)**	764 (88%)	1002 (81%)
**# ≥15-year-olds enumerated**	1235	1779
**# ≥ 15-year-olds surveyed (%)**	1033 (84%)	1485 (83%)
**#HH with improved drinking water source (%)**	402 (78%)	658 (85%)
**# HH with drinking water source within 30 minutes (%)**	507 (98%)	771 (100%)
**# HH with access to an improved latrine (%)**	418 (81%)	603 (78%)

**Table 2:** Baseline and impact survey data by evaluation unit.

		Tarawa		Kiritimati	
		Baseline	Impact	Baseline	Impact
**TF 1–9 years**	Adjusted prevalence (95% CI)	38.2 (35.7–41.5)	17.9 (14.7–21.8)	28 (24–35)*	11.5 (8.6–14.1)
**TI 1–9 years**	Adjusted prevalence (95% CI)		0.1 (0.0–0.4)		0.1 (0.0–0.3)
**TT ≥15 years**	Adjusted prevalence (95% CI)	0.8 (0.4–1.2)**	1.1 (0.67–1.7)	0.2 (0.1–0.3)*	0.41 (0.17–0.74)
	Unknown to health system; adjusted prevalence (95% CI)	n.d.	0.97 (0.56–1.5)	n.d.	0.39 (0.14–0.73)
**Infection 1–9 years**	Prevalence (95% CI)	27.4 (24.7–30.1)	3.3 (0.78–9.6)	24.0*†‡	0.96 (0.27–7.2)
**Seroprevalence 1–9 years (95% CI)**	MBA-Pgp3	51.2 (41.9–60.6)	31.4 (23.4–40.3)	46.8 (33.9–60.0)	30.2 (22.1–40.1)
	MBA CT694	48.8 (39.4–58.2)	30.7 (22.7–39.8)	46.1 (33.1–59.6)	29.6 (21.3–39.7)
	Pgp3 ELISA	50.6 (39.6–61.7)	29.5 (21.0–39.4)	44.4 (32.0–57.5)	27.3 (19.4–33.7)
	Pgp3 LFA	n.d.	28.0 (17.6–41.4)	n.d.	22.6 (14.6–33.7)

The superscripts * denote data taken from Reference 8 and ** from Reference 7. Superscripts † and ‡ denote infection data extrapolated from an incomplete dataset as described in Reference 8 and measured using droplet digital PCR. CI, confidence interval; n.d., not done; MBA, multiplex bead assay; ELISA, enzyme-linked immunosorbent assay; LFA, lateral flow assay; TF, trachomatous inflammation-follicular; TT, trachomatous trichiasis. Dried blood spot samples from baseline surveys were retested at the same time as samples from impact surveys.

In Kiritimati, there were 1033 persons aged ≥15 years examined for TT (Table 1). Ten had trichiasis, of whom six had trichiasis in both eyes; 1 had post-operative trichiasis. 9 (90%) of the 10 were unknown to the health system. The age-and-gender-adjusted trichiasis prevalence was 0.41% (95% CI 0.17%–0.74%) and the age- and gender-adjusted prevalence of trichiasis unknown to the health system was 0.39% (95% CI .14%–0.73%, Table 2). 70% of people with trichiasis (7/10) also had TS. Most trichiasis cases (7/10) were in women.

In Tarawa, 1485 persons aged ≥15 years were examined for TT (Table 1). Twenty-five had trichiasis, of which 16 were bilateral cases; 2 had post-operative trichiasis. 22 (88%) of these were unknown to the health system. The age-and-gender-adjusted trichiasis prevalence was 1.15% (95% CI 0.67%–1.74%) and the age- and gender- adjusted prevalence of trichiasis unknown to the health system was 0.97% (95% CI 0.56%–1.49%, Table 2). 76% of people with trichiasis (19/25) also had TS. Most trichiasis cases (18/25) were in women.

### Comparison of baseline and TIS serology data

**Fig 3:**
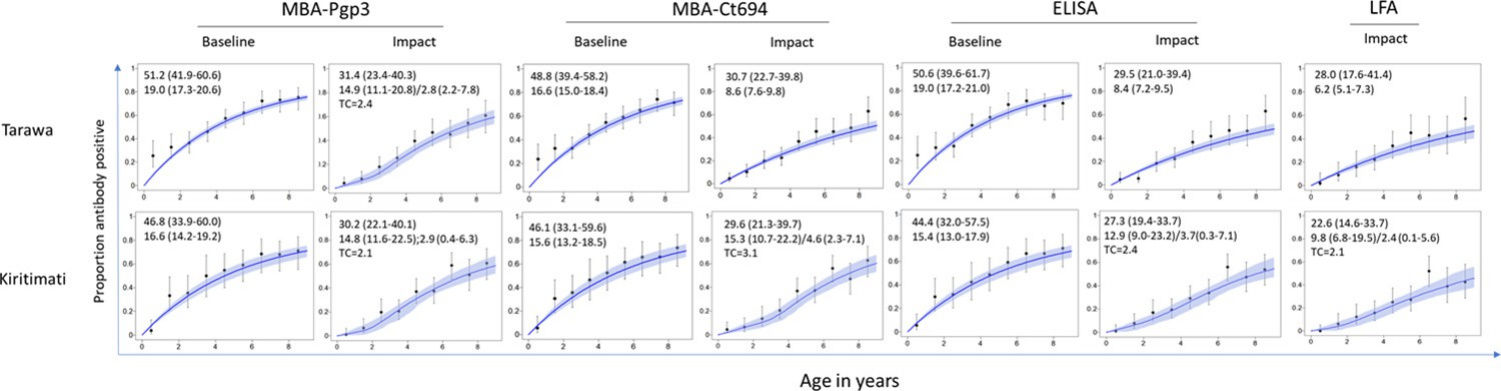
Change in serological indicators in Tarawa and Kiritimati Island before and after mass drug administration by multiple tests. The proportion seropositive (y-axis) by age (x-axis) from baseline and impact surveys measured by MBA (multiplex bead assay) for Pgp3 and CT694 as indicated, ELISA (enzyme-linked immunosorbent assay) for Pgp3, and LFA (lateral flow assay) for Pgp3. Data were modeled as per the methods, and 95% credible intervals are in the shaded region. Graphs were created using R version 3.6.3 (https://www.r-project.org/). The top number in the upper left corner is the age-adjusted seroprevalance (95% confidence intervals) and the bottom number is the seroconversion rate (SCR) per 100 children (95% credible interval) and the second number (for graphs generated with model 2) is the SCR after the time of change. TC = time of change. The LFA was not conducted on blood specimens collected during baseline surveys due to insufficient sample.

**Fig 4:**
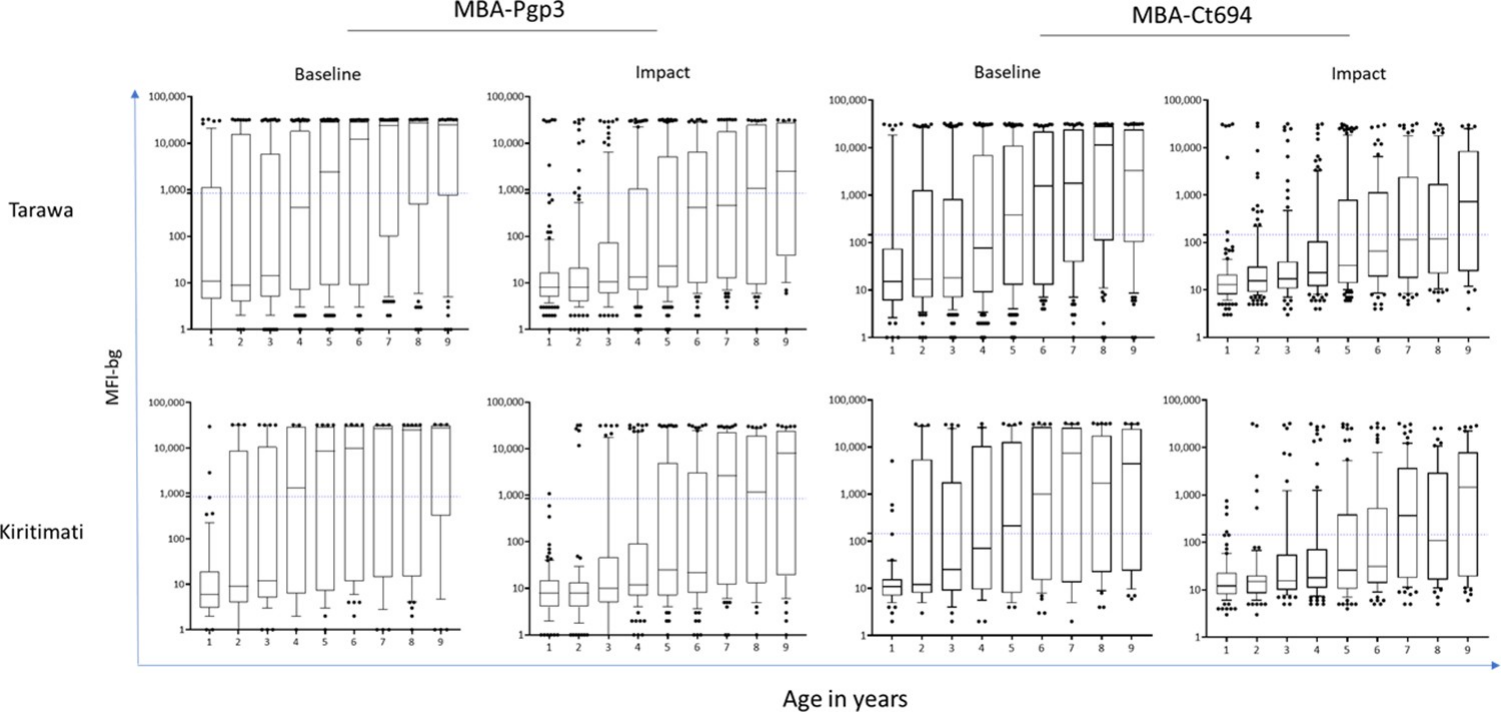
Change in antibody levels at baseline and trachoma impact survey (TIS) MBA. Semi-quantitative data obtained from the multiplex bead assay (MBA) and reported as the median fluorescence intensity with the background subtracted out (MFI-bg, y-axis) stratified by year of age by MBA. Data show interquartile range (IQR,10–90%), with median in box; dots represent values outside IQR.

**Fig 5:**
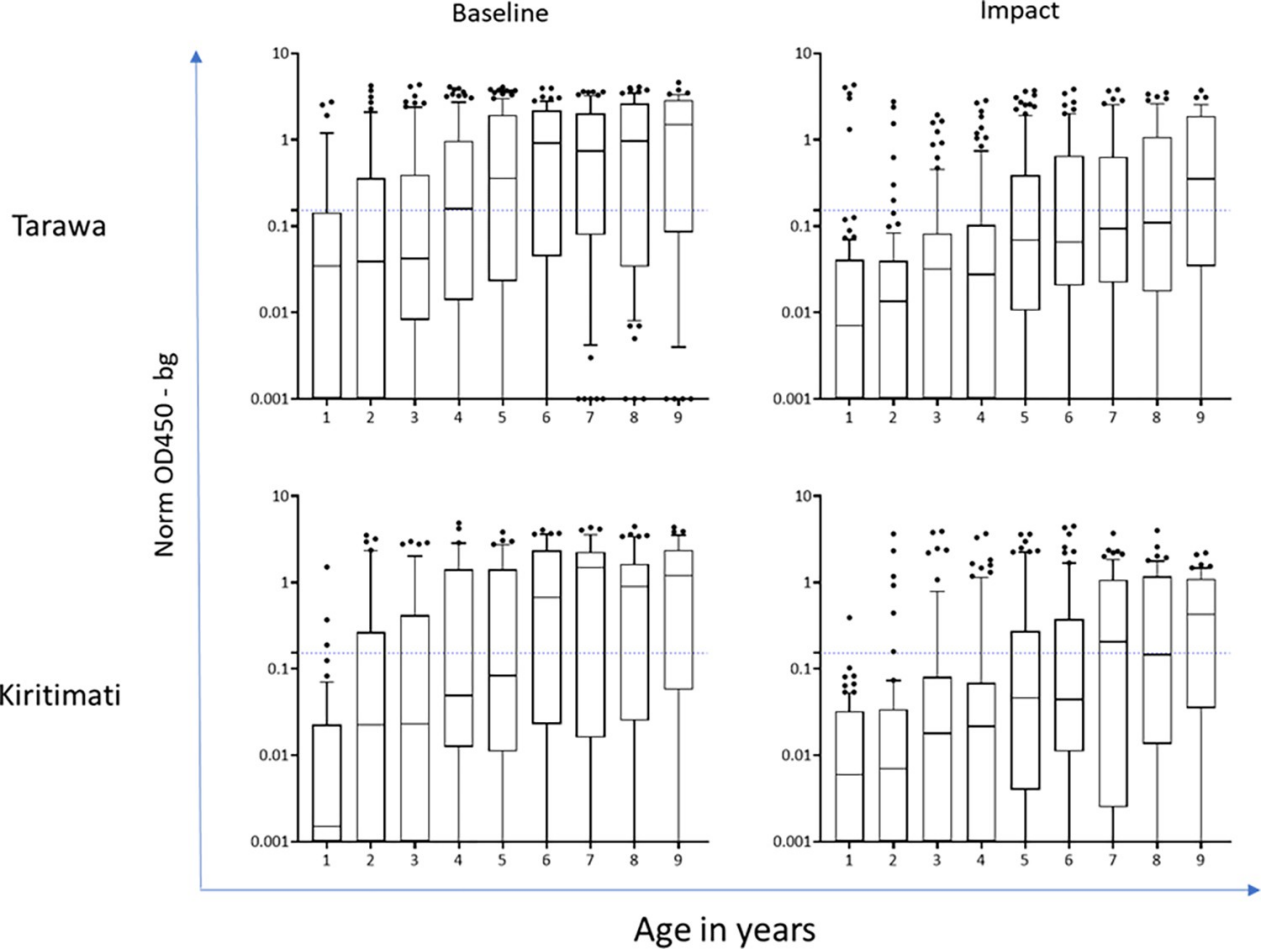
Antibody levels at baseline vs trachoma impact survey by ELISA. Data show the intensity of linked immunosorbent assay (ELISA) stratified by year of age. Y-axis shows optical density at 450 nm (OD450) with background subtracted (OD450-bg). Box and whiskers plots show the median and 10–90 percentile, with outliers shown as individual data points. Horizontal blue dotted line shows the assay cutoff. **** p < 0.05.

Seroprevalence estimates for anti-*C*. *trachomatis* antibodies in 1–9-year-olds were lower at TIS than at baseline by all tests in both EUs (Table 2, Fig 3). Model 2 (fixed time point) was a better fit for all assays in Kiritimati at TIS and Pgp3 MBA in Tarawa at TIS. The estimated time of change ranged from 2.1 to 3.1 years. The λc, representing the SCR following the point of change (i.e. in younger age ranges) ranged from 2.4 to 3.1 (Table 3). Model 1 (single rate of transmission) was a better fit for all assays at baseline on both Tarawa and Kiritimati and for Ct694 MBA, Pgp3 ELISA and Pgp3 LFA at TIS on Tarawa. SCRs were lower at TIS than at baseline for Ct694 MBA, Pgp3 ELISA and Pgp3 MBA (Table 3). Results from both models for each assay, island, time point, and model diagnostics are shown in S1 Table. In both Tarawa and Kiritimati, seroprevalence and SCR estimates were similar (overlapping CIs) whether using MBA, ELISA, or LFA (Tables 2 and 3, and Fig 4), despite a consistent trend of lower estimates produced by LFA compared to MBA. The overall seroprevalence in 1–3-year-olds was significantly lower at TIS than baseline by MBA (Pgp3 and Ct694) and ELISA (Table 4). There was evidence of lower median antibody levels at TIS than at baseline when measured using either MBA (Pgp3 and CT694, Fig 4) or ELISA (Pgp3, Fig 5).

**Table 3:** Serocatalytic modeling results.

Island	Timepoint	Test	Model	λT	(95% CrI)	λc	(95% CrI)	γ	(95% CrI)	p (rho)	(95% CrI)	time_c	(95% CrI)
Kiritimati	Baseline	MBA Pgp3	Model 1	16.6	(14.2–19.1)	-	-	-	-	2.6	(2.0–3.2)	-	-
		MBA Ct694	Model 1	15.6	(13.2–18.5)	-	-	-	-	1.7	(1.3–2.1)	-	-
		ELISA	Model 1	15.4	(13.0–17.9)	-	-	-	-	2.6	(2.0–3.2)	-	-
Kiritimati	Impact	MBA Pgp3	Model 2	14.8	(11.6–22.5)	2.9	(0.4–6.3)	0.2	(0.03–0.4)	2.7	(2.1–3.3)	2.1	(1.2–4.2)
		MBA Ct694	Model 2	15.3	(10.7–22.2)	4.6	(2.3–7.1)	0.3	(0.2–0.6)	1.7	(1.3–2.1)	3.1	(1.7–4.8)
		ELISA	Model 2	12.9	(9.0–23.2)	3.7	(0.3–7.1)	0.3	(0.03–0.6)	2.6	(2.0–3.2)	2.4	(0.9–5.4)
		LFA	Model 2	9.8	(6.8–19.5)	2.4	(0.1–5.6)	0.2	(0.02–0.6)	2.6	(2.0–3.2)	2.1	(0.8–5.5)
Tarawa	Baseline	MBA Pgp3	Model 1	19.0	(17.3–20.6)	-	-	-	-	2.5	(2.1–2.9)	-	-
		MBA Ct694	Model 1	16.6	(15.0–18.4)	-	-	-	-	1.7	(1.3–2.1)	-	-
		ELISA	Model 1	19.0	(17.2–21.0)	-	-	-	-	2.3	(1.9–2.7)	-	-
Tarawa	Impact	MBA Pgp3	Model 2	14.9	(11.1–20.8)	4.8	(2.2–7.8)	0.3	(0.1–0.5)	2.6	(2.0–3.2)	2.4	(1.1–4.2)
		MBA Ct694	Model 1	8.6	(7.6–9.8)	-	-	-	-	1.7	(1.3–2.1)	-	-
		ELISA	Model 1	8.4	(7.2–9.5)	-	-	-	-	2.6	(2.0–3.1)	-	-
		LFA	Model 1	8.0	(6.6–9.4)	-	-	-	-	2.6	(2.0–3.1)	-	-

Crl = credible interval; MBA = multiplex bead assay; LFA = lateral flow assay; λT = rate of seroconversion due to exposure to trachoma; λc rate of seroconversion due to exposure to trachoma, following the identified fixed point at which transmission intensity changed (time_c) p = rate of sero-reversion; γ = proportional decline in transmission at time_c or over time; time_c = time of change in transmission

**Table 4:** Seroprevalence in 1–3-year-olds by assay for baseline and trachoma impact surveys in Kiritimati Island and Tarawa.

	Kiritimati Island			Tarawa		
	Baseline	Impact	% change	Baseline	Impact	% change
**MBA Pgp3**	22.6 (16.4–30.3)	8.2 (5.3–12.4)	-64.0 (-39.4 –-79.2)	32.8 (27.1–39.0)	9.5 (6.9–12.9)	-71.0 (-58.6 –-80.1)
**MBA Ct694**	22.6 (16.4–30.3)	7.7 (4.9–11.9)	-65.9 (-42.1 –-80.6)	30.6 (25.1–36.8)	10.9 (8.1–14.5)	-64.5 (-50.0 –-75.3)
**ELISA**	21.3 (15.3–28.7)	7.9 (5.1–12.1)	-62.9 (-36.7 –-79.0)	30.8 (24.3–38.1)	8.8 (6.1–12.6)	-71.3 (-56.3 –-81.7)

MBA = multiplex bead assay; 95% confident intervals in parenthesis

### Infection prevalence at TIS

Of 1741 total swab containers received at CDC, 1548 swabs were tested and included in the final dataset. 150 swabs could not be linked to demographic data, 16 swabs were associated with multiple sets of demographic data, 4 swabs had duplicate IDs, 22 swabs had invalid tests, and 1 tube had no swab in it. The age-adjusted prevalence of infection in 1–9-year-olds at TIS was 3.3% (95% CI 0.78–9.6%) in Tarawa and 0.96% (95% CI 0.27–7.2%) in Kiritimati (Table 2). Of the swabs taken at TIS that had a positive PCR result, 68% (23/34) were from children with TF, 100% were from children with a positive MBA (30/30, all positive for antibodies to both Pgp3 and CT694) or LFA (24/24) and 96% were from children with a positive ELISA (27/28).

## Discussion

We report here data on three indicators for trachoma–TF, infection and antibody–at a TIS conducted after two (rather than the recommended three) annual rounds of MDA in two EUs in Kiribati [23]. While TF prevalence remained well above the 5% threshold taken as a stop-MDA signal, there was still a >50% decrease in TF prevalence at TIS compared to baseline in each EU (Table 2). There were also noticeable decreases in infection and serology indicators at TIS compared with baseline. By contrast, TT prevalence remained relatively constant. The TIS included data on TT unknown to the health system, one of the criteria for elimination as a public health problem. In each EU, the majority of TT cases were unknown to the health system, indicating that TT case-finding and provision of surgery to cases may need strengthening.

We saw changes in overall seroprevalence, SCR, and intensity of antibody response after two rounds of MDA in both EUs. There are limited population-level serological data before and after treatment for ocular *C*. *trachomatis* infection, so our work here provides a glimpse into changes in antibody profiles following MDA. The only other population-based dataset identifiable in literature published prior to August 2022 that compared serology before and after MDA is from Nepal; it showed little detectable antibody in 1–9-year-olds after MDA, but 10 years had elapsed between baseline and post-MDA sampling [24], compared with 2–3 years in these EUs. It is not surprising to see high antibody prevalence after two rounds of MDA in Kiribati, as we anticipate antibody responses to endure in sites with high active trachoma prevalence due to the presence of long-lived plasma cells that can secrete antibodies in the absence of restimulation. Studies in Kongwa, the United Republic of Tanzania, following children longitudinally, suggest seroreversion occurs at rates of about 6% per year in low prevalence areas (TF approximately 5% [25]) but was not observed in a high prevalence (TF >40%) community after 6 months [26]. In the current study, we also observed that correlates of antibody level, the absolute MFI-bg and OD-bg values, waned at population level after MDA. Though only a few studies have been completed, a picture is now emerging of population-based antibody prevalence in children decreasing slowly but steadily, with more rapid changes likely being seen in populations with lower baseline ocular *C*. *trachomatis* transmission intensities than in populations with higher baseline transmission intensities. The two EUs in Kiribati belong to the latter category.

While two rounds of MDA had a moderate impact on TF and antibody prevalence in each EU, there was a >8-fold decrease in infection prevalence in Tarawa, from 27.4% at baseline to 3.3% at TIS. Similar stark decreases in infection prevalence were detected in the United Republic of Tanzania [27,28] and Ethiopia [29] after one or two rounds of MDA, with two of these studies conducted in communities with starting infection prevalence >60% [28,29]. By contrast, communities in Senegal and Gambia with lower infection prevalence (approximately 3%) showed no decrease in infection prevalence after a single round of MDA [30]. It is not clear if the impact of 1–2 rounds of MDA is related to starting infection prevalence, with a greater impact in higher prevalence settings, or to the variety of strains circulating in the community [30]; it is also more difficult to reliably detect prevalence changes when the starting prevalence is low. However, because there were different in testing platforms used at baseline and TIS, the baseline survey had a limited infection dataset, these changes should be approached with caution.

A secondary outcome of this study was a comparison of different platforms for anti-Pgp3 serological testing. We continue to evaluate multiple platforms because each test has certain advantages: the LFA as a low-cost, low-tech test, the ELISA as a standard serological assay platform with semi-quantitative readouts, and the MBA as a mechanism to conduct integrated serosurveillance. The seroprevalence estimated using LFA was slightly lower than that estimated using MBA, though the difference was not statistically significant; otherwise there was no difference in population-level seroprevalence or SCR when using MBA, ELISA, or LFA. We have seen a similar difference in other settings [31] and believe it is due to a lower analytical limit of detection in the LFA than in the MBA. In this study, the 47 specimens that were positive by MBA but negative by LFA had a lower MFI-BG level than specimens positive by both tests, suggesting less antibody per specimen in those with discrepant results. A lower seroprevalence was also observed using LFA in comparison to the ELISA, with 32 samples positive by ELISA but not LFA; the specimens that were positive by ELISA but negative by LFA had lower OD values than double-positive samples. Both the MBA and ELISA have a signal amplification step as part of the secondary antibody binding reaction that is not present in the LFA. The lower analytical sensitivity of the LFA may have a larger effect in low-prevalence settings. This should be a consideration in test selection or sample size calculation. Overall, we saw measurable decreases in the population-level serological markers of seroprevalence and SCR in a relatively short time frame, using different testing platforms, associated with just two rounds of MDA, in a population that had high baseline ocular *C*. *trachomatis* transmission intensity. These data will be useful to understand population-level antibody half-life to better understand if and how serological testing can be used to support trachoma programs [11].

Our study has several limitations. There were not enough remaining DBSs to test baseline specimens by LFA to get robust prevalence estimates. Infection data from the TIS were generated using different instruments than at baseline, and on Kiritimati, not all children were able to be tested for infection at baseline [8]. Therefore, despite seemingly large decreases in infection prevalence after two rounds of MDA, these data should be approached with some caution. The TIS was conducted after two rounds of MDA rather than three rounds as per usual WHO recommendations, and so there may not have been enough time to observe more dramatic changes in serological measures, but we nevertheless observed 40–60% decreases in seroprevalence and SCR.

Despite the decreases in all infection-related metrics–TF, infection, and serology–after two rounds of MDA, the EUs evaluated here did not reach the target of 5% TF prevalence in 1–9-year-olds to discontinue MDA. An early TIS was conducted in Kiribati on the rationale that two high-coverage rounds of azithromycin might be sufficient to reach the 5% TF threshold owing to a relatively small, highly geographically constrained populations with universal close access to sea water for face washing found in Kiribati. The authors do not advocate doing early impact surveys as a routine. Additionally, TT prevalence at TIS remained >0.2% in ≥15-year-olds, indicating a need to continue surgical services.

### Disclaimer

The authors alone are responsible for the views expressed in this article and they do not necessarily represent the views, decisions or policies of the institutions with which they are affiliated.

## Supporting information

S1 Table Outputs and diagnostics from serocatalytic modeling assuming either a single, constant transmission rate (Model 1) or a single point of change in rate of transmission (Model 2). Each model was run with each dataset to determine the best-fitting model for each dataset. Data from the best-fitting models are reported in Table 3.
(DOCX)
