# Supplementary material for: Changes in trachoma indicators in Kiribati with two rounds of azithromycin mass drug administration, measured in serial population-based surveys
Source: PLoS Negl Trop Dis. 2023 Jul 7;17(7):e0011441. doi: 10.1371/journal.pntd.0011441 (PMC10355439; doi:10.1371/journal.pntd.0011441)
Supplement: S1 Table — Each model was run with each dataset to determine the best-fitting model for each dataset. Data from the best-fitting models are reported in Table 3. (DOCX) [file pntd.0011441.s001.docx]

| Island | Timepoint | Test | Model | λ0 | (95% CrI) | GR | GR Upper | ESS | λc | (95% CrI) | GR | GR Upper | ESS | γ | (95% CrI) | GR | GR Upper | ESS | p (rho) | (95% CrI) | GR | GR Upper | ESS | time_c | (95% CrI) | GR | GR Upper | ESS | DIC |
| --- | --- | --- | --- | --- | --- | --- | --- | --- | --- | --- | --- | --- | --- | --- | --- | --- | --- | --- | --- | --- | --- | --- | --- | --- | --- | --- | --- | --- | --- |
| Kiritimati | Baseline | MBA Pgp3 | *Model 1 | 16.6 | (14.2-19.1) | 1.01 | 1.03 | 835 | - | - | - | - | - | - | - | - | - | - | 2.6 | (2.0-3.2) | 1 | 1 | 4467 | - | - | - | - | - | 453 |
|  |  | MBA Pgp3 | Model 2 | 42.1 | (16.7-89.5) | 2.94 | 13.42 | 362 | 16 | (2.1-18.9) | 2 | 4.52 | 747.19 | 0.32 | (0.1-0.9) | 1.76 | 3.77 | 219 | 2.6 | (2.0-3.1) | 1.03 | 1.11 | 502 | 9.7 | (0.54-24.4) | 3.25 | 13.66 | 105.961 | 446 |
|  |  | MBA Ct694 | *Model 1 | 15.6 | (13.2-18.5) | 1.02 | 1.06 | 623 | - | - | - | - | - | - | - | - | - | - | 1.7 | (1.3-2.1) | 1 | 1.01 | 3324 | - | - | - | - | - | 455 |
|  |  | MBA Ct694 | Model 2 | 18.6 | (14.6-30.3) | 2.54 | 6.97 | 104 | 13.7 | (0.70-17.5) | 1.14 | 1.39 | 1223 | 0.64 | (0.04-1) | 2.03 | 3.58 | 139 | 1.7 | (1.3-2.1) | 1 | 1.01 | 567 | 2.7 | (0.2-15.3) | 2.03 | 4.19 | 206 | 453 |
|  |  | ELISA | *Model 1 | 15.4 | (13.0-17.9) | 1 | 1 | 885 | - | - | - | - | - | - | - | - | - | - | 2.6 | (2.0-3.2) | 1 | 1 | 4313 | - | - | - | - | - | 471 |
|  |  | ELISA | Model 2 | 20.6 | (14.8-34.5) | 3.31 | 8.06 | 34 | 14.8 | (9.3-17.7) | 1.11 | 1.26 | 773 | 0.7 | (0.4-1.0) | 2.71 | 6.44 | 80 | 2.6 | (2.0-3.2) | 1.01 | 1.01 | 704 | 12.1 | (0.4-78.2) | 1.66 | 2.98 | 84 | 470 |
| Kiritimati | Impact | MBA Pgp3 | Model 1 | 9 | (7.7-10.3) | 1 | 1 | 1891 | - | - | - | - | - | - | - | - | - | - | 2.5 | (2.0-3.2) | 1 | 1 | 3576 | - | - | - | - | - | 689 |
|  |  | MBA Pgp3 | *Model 2 | 14.8 | (11.6-22.5) | 1.01 | 1.02 | 547 | 2.9 | (0.4-6.3) | 1.01 | 1.05 | 420 | 0.2 | (0.03-0.4) | 1.01 | 1.05 | 462.5 | 2.7 | (2.1-3.3) | 1.02 | 1.05 | 397 | 2.1 | (1.2-4.2) | 1.01 | 1.03 | 476 | 670 |
|  |  | MBA Ct694 | Model 1 | 8.5 | (7.4-9.8) | 1 | 1 | 1453 | - | - | - | - | - | - | - | - | - | - | 1.7 | (1.3-2.1) | 1 | 1.01 | 2673 | - | - | - | - | - | 686 |
|  |  | MBA Ct694 | *Model 2 | 15.3 | (10.7-22.2) | 1.01 | 1.02 | 909 | 4.6 | (2.3-7.1) | 1 | 1.01 | 993 | 0.3 | (0.2-0.6) | 1.03 | 1.04 | 546.7 | 1.7 | (1.3-2.1) | 1 | 1 | 1371 | 3.1 | (1.7-4.8) | 1.01 | 1.01 | 1046 | 672 |
|  |  | ELISA | Model 1 | 7.9 | (6.7-9.2) | 1 | 1.01 | 1838 | - | - | - | - | - | - | - | - | - | - | 2.5 | (2.0-3.1) | 1 | 1.01 | 3289 | - | - | - | - | - | 653 |
|  |  | ELISA | *Model 2 | 12.9 | (9.0-23.2) | 1.01 | 1.03 | 632 | 3.7 | (0.3-7.1) | 1.01 | 1.04 | 763 | 0.3 | (0.03-0.6) | 1.02 | 1.04 | 615 | 2.6 | (2.0-3.2) | 1.01 | 1.05 | 1384 | 2.4 | (0.9-5.4) | 1.01 | 1.05 | 790 | 640 |
|  |  | LFA | Model 1 | 6.2 | (5.1-7.3) | 1.01 | 1.02 | 2036 | - | - | - | - | - | - | - | - | - | - | 2.6 | (2.0-3.1) | 1 | 1 | 3552 | - | - | - | - | - | 471 |
|  |  | LFA | *Model 2 | 9.8 | (6.8-19.5) | 1 | 1 | 514 | 2.4 | (0.1-5.6) | 1.01 | 1.04 | 600 | 0.2 | (0.02-0.6) | 1.03 | 1.09 | 568.6 | 2.6 | (2.0-3.2) | 1.03 | 1.1 | 1093 | 2.1 | (0.8-5.5) | 1 | 1 | 634 | 462 |
| Tarawa | Baseline | MBA Pgp3 | *Model 1 | 19 | (17.3-20.6) | 1 | 1.01 | 806 | - | - | - | - | - | - | - | - | - | - | 2.5 | (2.1-2.9) | 1 | 1 | 1945 | - | - | - | - | - | 2371 |
|  |  | MBA Pgp3 | Model 2 | 28.6 | (25.2-32.5) | 2.15 | 9.67 | 77 | 18.5 | (16.0-20.4) | 1.47 | 3.18 | 456 | 0.65 | (0.6-0.7) | 1.97 | 3.9 | 53 | 2.5 | (2.1-2.9) | 1.01 | 1.01 | 1056 | 10.1 | (4.1-20.0) | 1.92 | 7.81 | 37 | 2371 |
|  |  | MBA Ct694 | *Model 1 | 16.6 | (15.0-18.4) | 1.01 | 1.05 | 977 | - | - | - | - | - | - | - | - | - | - | 1.7 | (1.3-2.1) | 1.01 | 1.02 | 2961 | - | - | - | - | - | 1121 |
|  |  | MBA Ct694 | Model 2 | 25.6 | (16.7-88.3) | 1.68 | 4.86 | 16 | 16.5 | (14.9-18.2) | 1.02 | 1.03 | 815 | 0.64 | (0.2-1.0) | 1.46 | 2.34 | 42 | 1.7 | (1.3-2.0) | 1.01 | 1.01 | 472 | 48.6 | (6.6-88.4) | 1.94 | 3.91 | 111 | 1121 |
|  |  | ELISA | *Model 1 | 19 | (17.2-21.0) | 1.01 | 1.01 | 742 | - | - | - | - | - | - | - | - | - | - | 2.3 | (1.9-2.7) | 1 | 1 | 1620 | - | - | - | - | - | 1873 |
|  |  | ELISA | Model 2 | 24.1 | (18.3-39.0) | 1.73 | 4.19 | 43 | 18.4 | (6.4-20.6) | 1.04 | 1.12 | 563 | 0.7 | (0.3-1.0) | 1.75 | 3.24 | 57 | 2.4 | (1.9-2.8) | 1.05 | 1.15 | 969 | 10.4 | (0.2-34.9) | 1.4 | 2.4 | 45 | 1870 |
| Tarawa | Impact | MBA Pgp3 | Model 1 | 9.2 | (8.0-10.4) | 1.01 | 1.05 | 1987 | - | - | - | - | - | - | - | - | - | - | 2.5 | (2.0-3.1) | 1 | 1 | 3254 | - | - | - | - | - | 895 |
|  |  | MBA Pgp3 | *Model 2 | 14.9 | (11.1-20.8) | 1 | 1.01 | 1683 | 4.8 | (2.2-7.8) | 1 | 1.01 | 1733 | 0.3 | (0.1-0.5) | 1 | 1 | 1323.5 | 2.6 | (2.0-3.2) | 1 | 1.01 | 2462 | 2.4 | (1.1-4.2) | 1.04 | 1.05 | 1532 | 880 |
|  |  | MBA Ct694 | *Model 1 | 8.6 | (7.6-9.8) | 1 | 1.01 | 1450 | - | - | - | - | - | - | - | - | - | - | 1.7 | (1.3-2.1) | 1 | 1 | 2550 | - | - | - | - | - | 896 |
|  |  | MBA Ct694 | Model 2 | 13.3 | (9.1-36.4) | 1.06 | 1.14 | 263 | 5.7 | (3.5-9.3) | 1.04 | 1.11 | 272 | 0.4 | (0.2-0.8) | 1.07 | 1.21 | 271 | 1.7 | (1.4-2.1) | 1.02 | 1.07 | 416 | 2.8 | (0.9-80.1) | 1.04 | 1.05 | 187 | 889 |
|  |  | ELISA | *Model 1 | 8.4 | (7.2-9.5) | 1 | 1.01 | 1935 | - | - | - | - | - | - | - | - | - | - | 2.6 | (2.0-3.1) | 1 | 1 | 3437 | - | - | - | - | - | 707 |
|  |  | ELISA | Model 2 | 13.7 | (9.8-21.1) | 1.18 | 1.41 | 528 | 4.3 | (1.8-7.4) | 1.04 | 1.13 | 643 | 0.3 | (0.1-0.6) | 1.01 | 1.01 | 489.4 | 2.6 | (2.0-3.2) | 1 | 1.01 | 1153 | 2.4 | (1.4-4.7) | 1.15 | 1.39 | 582 | 696 |
|  |  | LFA | *Model 1 | 8 | (6.6-9.4) | 1.01 | 1.01 | 1578 | - | - | - | - | - | - | - | - | - | - | 2.6 | (2.0-3.1) | 1 | 1.01 | 3913 | - | - | - | - | - | 409 |
|  |  | LFA | Model 2 | 29 | (8.2-121.2) | 1.1 | 1.28 | 80 | 8 | (6.5-9.7) | 1.19 | 1.56 | 4247 | 0.3 | (0.1-0.9) | 1.03 | 1.09 | 374 | 2.6 | (2.0-3.2) | 1 | 1.01 | 2876 | 56.2 | (28.3-87.8) | 1.55 | 2.52 | 43 | 409 |

| Supplemental table 1: Serocatalytic model diagnostics | | | | | |  |  |  |  |  |  |  |  |  |  |  |  |  |  |  |  |  |  |  |  |  |  |  |  |
| --- | --- | --- | --- | --- | --- | --- | --- | --- | --- | --- | --- | --- | --- | --- | --- | --- | --- | --- | --- | --- | --- | --- | --- | --- | --- | --- | --- | --- | --- |
|  |  |  |  |  |  |  |  |  |  |  |  |  |  |  |  |  |  |  |  |  |  |  |  |  |  |  |  |  |  |
| *Selected model, based on lower DIC or lack of autocorrelation | | | | | |  |  |  |  |  |  |  |  |  |  |  |  |  |  |  |  |  |  |  |  |  |  |  |  |
| Model parameters λT, λc, and p were scaled *100 for ease of interpretation | | | | | | | |  |  |  |  |  |  |  |  |  |  |  |  |  |  |  |  |  |  |  |  |  |  |
| CrI: Credible Intervals; MBA: multiplex bead assay; LFA: lateral flow assay; GR: Gelman-Rubin statistic; ESS: Effective sample size; DIC: Deviance information criterion; λT: rate of seroconversion due to exposure to trachoma; λc: rate of seroconversion due to exposure to trachoma, following the identified fixed time point at which transmission intensity changed (time_c); ρ: rate of sero-reversion;  γ: proportional decline in transmission at time_c or over time. | | | | | | | | | | | | | | | | | | | | | | | | | | | | | |
